# Probing driving forces for binding between nanoparticles and amino acids by saturation-transfer difference NMR

**DOI:** 10.1038/s41598-020-69185-7

**Published:** 2020-07-23

**Authors:** Hui Xu, Leah B. Casabianca

**Affiliations:** Department of Chemistry, Clemson University, Clemson, SC 29634 USA

**Keywords:** Chemistry, Physical chemistry

## Abstract

As nanotechnology becomes increasingly used in biomedicine, it is important to have techniques by which to examine the structure and dynamics of biologically-relevant molecules on the surface of engineered nanoparticles. Previous work has shown that Saturation-Transfer Difference (STD)-NMR can be used to explore the interaction between small molecules, including amino acids, and the surface of polystyrene nanoparticles. Here we use STD-NMR to further explore the different driving forces that are responsible for these interactions. Electrostatic effects are probed by using zwitterionic polystyrene beads and performing STD-NMR experiments at high, low, and neutral pH, as well as by varying the salt concentration and observing the effect on the STD buildup curve. The influence of dispersion interactions on ligand-nanoparticle binding is also explored, by establishing a structure–activity relationship for binding using a series of unnatural amino acids with different lengths of hydrophobic side chains. These results will be useful for predicting which residues in a peptide are responsible for binding and for understanding the driving forces for binding between peptides and nanoparticles in future studies.

## Introduction

Nanoparticles are currently widely produced and applied in medical diagnostics and therapeutics. Examples include drug delivery, biosensors, and imaging contrast agents^1–3^. However, the specific effects of nanoparticles on biological systems are not always well known. Studies indicate that once the nanoparticles enter a biological system, they will be immediately surrounded by proteins^4–6^. Binding to the nanoparticles could change the conformation of proteins, resulting in new epitopes being exposed on the protein surface^7^. This could lead to unexpected reactions and toxicity. Having analytical techniques readily available for determining this protein structural change will allow us to understand and prevent the unexpected effects of nanoparticles in biological systems.

NMR is an attractive technique for observing conformational changes of proteins on nanoparticles due to the atomic-level structural information that NMR can provide and the non-destructive nature of this technique. Observing the structure of ligands on nanoparticle surfaces using solution-state NMR has difficulties, however. The main difficulty is the large size of nanoparticles which leads to slow tumbling in solution. This in turn leads to short nuclear *T*_2_ relaxation times, causing broad lines in the solution-state NMR spectrum. In some cases, the line broadening may become so severe that the lines are broadened into the baseline, making signals from the nanoparticle and strongly-bound ligands impossible to see. However, several NMR techniques have been used to analyze the structure of small molecule ligands and proteins on the surface of nanoparticles in solution^8,9^.

When ligands are in rapid exchange between the free and bound form, or have sufficient motion in the bound form to be observable, standard solution-state NMR techniques such as one-dimensional experiments, diffusion ordered spectroscopy (DOSY), Nuclear Overhauser Effect (NOE) or relaxation time measurements can be used to probe the structure and dynamics of ligand binding. For example, Oliva-Puigdomènech have used ^1^H NMR and DOSY to monitor dynamic ligand exchange between amines and carboxylic acids on Cu nanocrystals and found that the binding actually depends on the residual surface oxide, not the pristine Cu surface^10^. Earlier studies by the same group explored binding of phosphonic acids to CdSe quantum dots^11^, acids and bases interacting with metal oxide nanocrystals^12^, and binding site heterogeneity on CdSe nanocrystal surfaces^13–15^. Similar work using proton NMR to monitor competitive ligand exchange on CdSe and PbS nanocrystal surfaces has been done by the Dempsey group^16–18^. Glaria et al.^19^ found that capping ligands on Cu nanoparticles can be detected indirectly, through transferred NOEs (trNOE) to free ligands in solution. The Mattoussi group has combined ^1^H NMR, DOSY, and heteronuclear single quantum coherence (HSQC) to probe the composition of the organic coating layer on semiconductor quantum dots, to differentiate free and bound ligands, and to quantify the ligand density on quantum dots of different diameters^20,21^. Zhang et al.^22^ investigated the dynamics and morphology of polymers that are covalently linked to nanodiamonds through various NMR experiments and calculated the ratio of mobile and immobile nanoparticle-wrapping polymer segments. Wu and coworkers^23^ employed ^1^H chemical shifts, *T*_2_ relaxation times, peak widths, and integrals of ligand NMR signals to determine aspects of ligand structure on gold nanoparticles, such as packing density, headgroup motion, and the effect of surface curvature on ligand binding. Coelho et al.^24^ used NMR lineshape, DOSY, *T*_1_ relaxation times, and NOE measurements to characterize the loading of the anti-cancer drug Bortezomib (BTZ) into poly(ethylene glycol) (PEG)-functionalized gold nanoparticles. They discovered that two modes of binding are present: BTZ can associate with the PEG groups as well as bind to the gold nanoparticle surface through electrostatic interactions.

Other strategies involve either minimizing or using the line broadening induced when ligands bind to nanoparticle surfaces. Egner et al.^25^ showed that a 1% agarose gel could be used to stabilize nanoparticle suspensions and prevent sedimentation, allowing solution-state NMR studies of adsorption of small molecules on nanoparticle surfaces. De Roo et al.^26^ proposed using the line broadening of ligand resonances to understand solvent-ligand interactions. They found that the NMR lineshape of bound ligand resonances consists of homogeneous and heterogeneous broadening. The homogeneous broadening is correlated with the size of the nanoparticle, and the heterogeneous broadening reports on ligand-solvent interactions. More complete solvation of the ligands that are bound to the nanoparticle surface leads to a more uniform distribution of chemical environments for the bound ligands, and a reduced heterogeneous line broadening. Bartot et al.^27^ found that different isomers of ubiquitin had similar binding affinity to small nanoparticles, while larger nanoparticles separated the isomers based on binding affinity. Paramagnetic relaxation enhancement maps obtained from incorporating a paramagnetic metal center into lipid nanoparticles indicated that the two ubiquitin isomers bind to the larger nanoparticles with different binding regions. Brüschweiler and co-workers^28,29^ have used differential relaxation times to probe the residue-specific interactions between intrinsically-disordered proteins and silica nanoparticles with negatively-charged surfaces. The binding between intrinsically-disordered proteins and silica nanoparticles has been used to study protein dynamics on timescales that were previously un-reachable^30^.

In this and our previous work^31–34^, we use a ligand-detected NMR technique called Saturation-Transfer Difference (STD)-NMR^35,36^ to indirectly probe ligand binding on a nanoparticle surface. The STD-NMR experiment requires collecting two spectra, an off-resonance spectrum and an on-resonance spectrum. The off-resonance spectrum is acquired with saturation at a frequency where neither receptor/nanoparticle nor ligand/amino acid would resonate. The on-resonance spectrum is obtained by saturating at a frequency where only receptors resonate. The saturation will then spread to the entire nanoparticle by spin diffusion and will also reach ligand binding pockets. Ligands that bind the receptor then receive some transferred saturation. When these bound ligands dissociate from the receptor binding site, their peak intensity will decrease. The difference spectrum is then obtained by subtracting the on-resonance spectrum from the off-resonance spectrum. Ligands that do not interact with the receptor during the saturation time, on the other hand, have the same peak intensity in the on- and off-resonance spectra, so that in the difference spectrum, only peaks from binding ligands will show up. The binding can be quantified by the STD effect, which is defined as the STD difference intensity divided by the reference intensity^36^. An STD buildup curve can also be constructed, by plotting the STD effect as a function of saturation time. There are many factors that can affect the STD effect, including relaxation time of protons on both ligands and receptors, ligand:receptor ratio, and ligand rebinding during the saturation time^37^. Angulo et al.^38^ showed that the initial slope of the buildup curve is a more accurate way to measure the relative strength of binding than using STD effects at one particular saturation time, since this method can mitigate the ligand rebinding effects and the influence of different relaxation of protons during longer saturation times.

STD-NMR has been widely used to study the binding of ligands to biomacromolecules^39,40^ and even living cells^41^. Several studies have also appeared in the literature concerning using STD-NMR to study the binding between small molecule ligands and nanoparticles as well. Szczygiel et al.^42^ used STD NMR to characterize particle-dispersant interactions, including differentiating binding ligands from nonbinding ligands and determining the morphology of ligands on a nanoscale particle surface in situ. Hens et al.^43^ suggest that STD-NMR may have potential for studying proton-containing small molecule species adsorbed on the surface of colloidal nanocrystals. Suzuki et al.^44,45^ used STD-NMR to study the binding between a Ti-binding peptide and TiO_2_ or SiO_2_ nanoparticles.

In previous work^31^, we examined the binding between amino acids and polystyrene nanoparticles. We found significant STD effects for amino acids that have aromatic, positively-charged, or long aliphatic side chains, and attributed the binding between these amino acids and the polystyrene nanoparticles to pi–pi interactions, electrostatic interactions, and hydrophobic effects, respectively. Our results were similar to those found for amino acids interacting with the surface of silica nanoparticles^28^. In both cases, positively-charged amino acids were found to exhibit strong interactions with the negatively-charged surface. Amino acids with long hydrophobic chains were also seen to bind strongly to both silica and polystyrene nanoparticles. However, in the case of polystyrene nanoparticles, the aromatic amino acids also exhibit strong binding, whereas the aromatic amino acids do not bind strongly to silica nanoparticles, which lack aromatic groups. In the current work, we perform a series of experiments to test these hypotheses, and shed more light into the reasons for binding between small molecules (represented by amino acids in this case) and polystyrene nanoparticles.

In order to investigate the role of electrostatic effects, we performed STD-NMR experiments at different pH, and with aliphatic amine beads instead of carboxylate modified polystyrene beads. These aliphatic amine polystyrene beads are zwitterionic, with a high density of both amine and carboxylate groups on the surface. Based on zeta potential measurements, we found that these beads are positively charged at low pH, and negatively charged at near neutral and high pH. Varying the pH, therefore, allowed us to examine nanoparticle-amino acid interactions in which the two charges were equal, opposite, or one charged and one neutral. Additionally, we examined nanoparticle-amino acid binding under conditions of high and low salt concentration. High salt concentration is expected to shield positive and negative charges, and if electrostatic effects are the driving force for binding, then the STD effect is expected to decrease under high salt conditions.

To probe whether hydrophobic effects are primarily responsible for binding of long-chain aliphatic amino acids to polystyrene nanoparticle beads, we examined the STD effect for binding between polystyrene nanoparticles for several unnatural amino acids containing increasingly long aliphatic side chains.

The studies done in the current work provide more evidence for the various modes of binding to polystyrene nanoparticles that were previously proposed, and further validate the use of solution-state saturation-transfer difference NMR as a technique to study ligand binding to nanoparticle surfaces.

## Methods

Aliphatic amine polystyrene latex beads (2% w/v suspension in de-ionized water, 0.04 µm) were purchased from Thermo Fisher Scientific (Waltham, MA, USA). According to the manufacturer, the beads are zwitterionic with high density of amine and carboxyl groups on the surface. All amino acids were purchased from Sigma-Aldrich (St. Louis, MO, USA). Sodium hydroxide, sodium phosphate monobasic monohydrate (certified ACS, crystalline) and sodium phosphate dibasic heptahydrate (certified ACS, crystalline) and deuterium oxide (99.8 atom % D, for NMR, Acros Organics) were purchased from Fisher Scientific (Hampton, NH, USA). Phosphoric acid was purchased from Sigma Life Science (St. Louis, MO, USA). All reagents and solvents were used as received.

Zeta potential was measured on a Brookhaven NanoBrook Omni particle size and zeta potential analyzer (Holtsville, NY, USA) using Phase Analysis Light Scattering (PALS) zeta potential measurement mode. Each measurement was done in triplicate. The Smoluchowski zeta potential model was used to analyze the data^46^. Zeta potential measurements were made on samples of beads only in the absence of amino acids, in H_2_O with a phosphate buffer concentration of 10 mM or with samples in which the pH was adjusted by addition of 0.1 M HCl and 0.01 M HCl and NaOH solutions.

For NMR samples, 200 mM phosphate buffer in D_2_O was used to prepare samples at pH 2, 6 and 9. The actual pH values reported in Table 1 and Supplementary Table S1 in the supporting information are values that were directly measured by the pH probe, without any corrections for isotope effect. All amino acid concentrations were 35 mM and all weight percents of aliphatic amine beads were 0.21% w/v in the final samples. For the arginine sample described in Fig. 4 in which the pH was adjusted with 0.1 M HCl, the arginine concentration is slightly less than 35 mM. Tyrosine was not tested at any pH and glutamic acid and aspartic acid were not tested at pH 6, due to low solubility. The samples were transferred into 5 mm od NMR tubes (Norell inc, Morganton, NC, USA) for NMR measurements.

**Table 1:** Net ionic charge of amino acids at high, neutral, and low pH.

Amino acid	Actual pH	Net ionic charge	Actual pH	Net ionic charge	Actual pH	Net ionic charge
Trp	2.04	0.69	6.25	0.00	9.45	− 0.56
Phe	2.03	0.59	6.35	0.00	9.19	− 0.56
His	2.26	1.22	6.42	0.29	9.49	− 0.71
Arg	2.35	1.32	6.64	1.00	10.97	− 0.06
Lys	2.15	1.50	6.53	1.00	10.33	− 0.25
Met	2.00	0.59	6.27	0.00	9.38	− 0.67
Leu	2.00	0.68	6.28	0.00	9.83	− 0.64
Ile	2.02	0.63	6.31	0.00	9.76	− 0.59
Pro	2.05	0.44	6.30	0.00	10.42	− 0.47
Glu	1.87	0.66			9.77	− 1.61
Asp	1.88	0.53			9.90	− 1.63

In each case, the actual pH was measured for the final sample including buffer, polystyrene beads, and amino acid. The net ionic charge is calculated based on the literature pKa values of each amino acid (from the 2010 CRC Handbook of Chemistry and Physics) and the actual pH.

All NMR experiments were performed on a Bruker 500 MHz NEO NMR spectrometer with a BBO Prodigy nitrogen-cooled cryoprobe. One-dimensional proton experiments used a 12.5-µs 90° pulse with a 1-s recycle delay, 8 scans, 12 ppm spectral width and 3-s acquisition time. STD experiments were performed using the standard sequence, stddiffesgp, from the Bruker pulse sequence library.^35,36^ Off-resonance saturation was performed at 40 ppm and on-resonance saturation was performed at 12 ppm. No signal is present in the NMR spectra at 12 ppm, and control experiments for amino acid samples in the absence of polystyrene beads confirmed that saturation at 12 ppm did not disturb the ligand resonances. Saturation of the polystyrene beads was achieved by a train of Gaussian pulses of 50 ms each. 8 scans were collected in an interleaved manner for each on- and off-resonance spectrum. The STD experiment was acquired with a 3-s acquisition time and 12 ppm spectral width. Four dummy scans were collected at the beginning of each STD experiment. Saturation times and recycle delays were adjusted for the STD buildup time of each sample, and ranged from 7 to 15 s and 2 to 12 s for the recycle delays and saturation times, respectively. For both 1D ^1^H and STD experiments, the excitation sculpting with gradients water suppression sequence was used^47^. All experiments were done at 298 K. Bruker Topspin 4.0.5 and 4.0.6 software were used to process all NMR spectra. A custom-written MATLAB script was used to process phase corrected spectra to calculate peak integrals. All MATLAB operations were done with MATLAB R2018a software (MathWorks, Natick, MA, USA).

## Results and discussion

We have screened all 20 amino acids (with the exception of tyrosine, aspartic acid, and glutamic acid for reasons of low solubility) for binding to the surface of aliphatic amine nanoparticle polystyrene beads using the STD method. STD experiments were first performed for samples containing 35 mM amino acid and 0.21% w/v of polystyrene latex beads with a saturation time of 10 s. For those amino acid samples for which an STD effect was observable at a 10-s saturation time, the full STD buildup curve was constructed by performing experiments at several saturation times. The resulting STD buildup curve was then fit to an exponential buildup equation:

1 $$S\left(t\right)={S}_{max}\left(1-{e}^{-kt}\right),$$

where S(t) is the STD effect at time t, S_max_ is the maximum STD effect and k is a buildup curve. Best-fit values of S_max_ and k were determined for each buildup curve from a nonlinear least-squares fit. The initial slope of the STD buildup curve was then calculated as the derivative of Eq. (1) at time 0,

2 $$\frac{\partial S\left(t\right)}{\partial t}={S}_{max}\left({ke}^{-kt}\right),$$

3 $${\left.\frac{\partial S\left(t\right)}{\partial t}\right|}_{t=0}={S}_{max}\times k\times 1={S}_{max}\times k.$$

**Figure 1:**
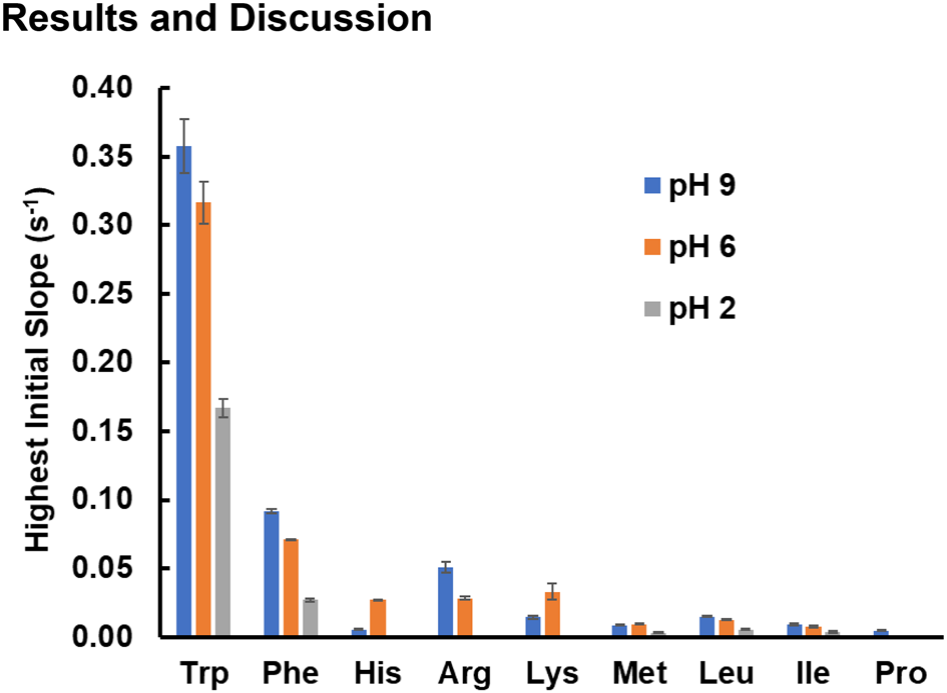
Initial slope of the STD buildup curve for the nine amino acids showing significant STD effects at high, neutral, and low pH. For amino acids with multiple proton peaks, the highest initial slope is shown in each case (the highest initial slope at high, neutral, and low pH may not necessarily represent the same proton). Amino acids that are not listed did not show appreciable STD effects with a 10-s saturation time. Exceptions are tyrosine at all pH values and aspartic acid and glutamic acid at pH 6, which were not tested due to low solubility.

For those amino acids that exhibited observable STD effects at a 10-s saturation time and for which an STD buildup curve was constructed, the highest initial slope of the buildup curve at low, neutral, and high pH is shown in Fig. 1. For amino acids with more than one proton peak, the value for the peak with the highest initial slope is shown. Error bars are propagated errors from the fit to S_max_ and k. These values and errors are also listed in the Supplementary Table S2.

**Figure 2:**
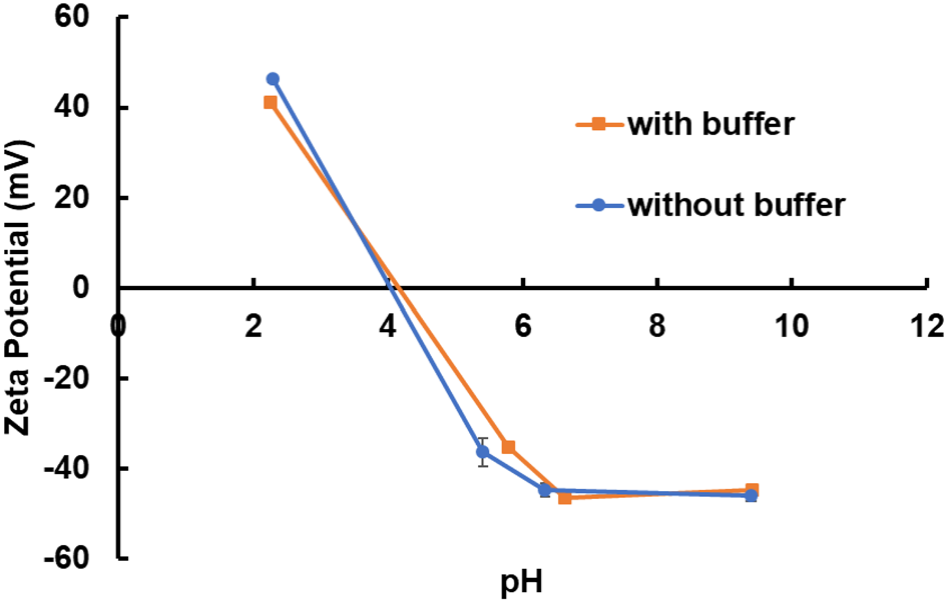
Zeta potential measurement for polystyrene nanoparticles at different pH. The pH was adjusted with 10 mM phosphate buffer. For pH adjusted without buffer, 0.1 M and 0.01 M HCl and NaOH solutions are used to achieve the target pH value.

From Fig. 1, the highest initial slopes at all pH values are for the aromatic amino acids tryptophan and phenylalanine. The next highest values of initial slope of the STD buildup curve listed in Fig. 1 are for histidine, arginine, and lysine, which are nominally positively charged at neutral pH. To explore the effect of pH in more detail, the zeta potential of the polystyrene beads at high, neutral, and low pH is shown in Fig. 2 and the net ionic charge of each amino acid at the various pH values are listed in Table 1.

**Figure 3:**
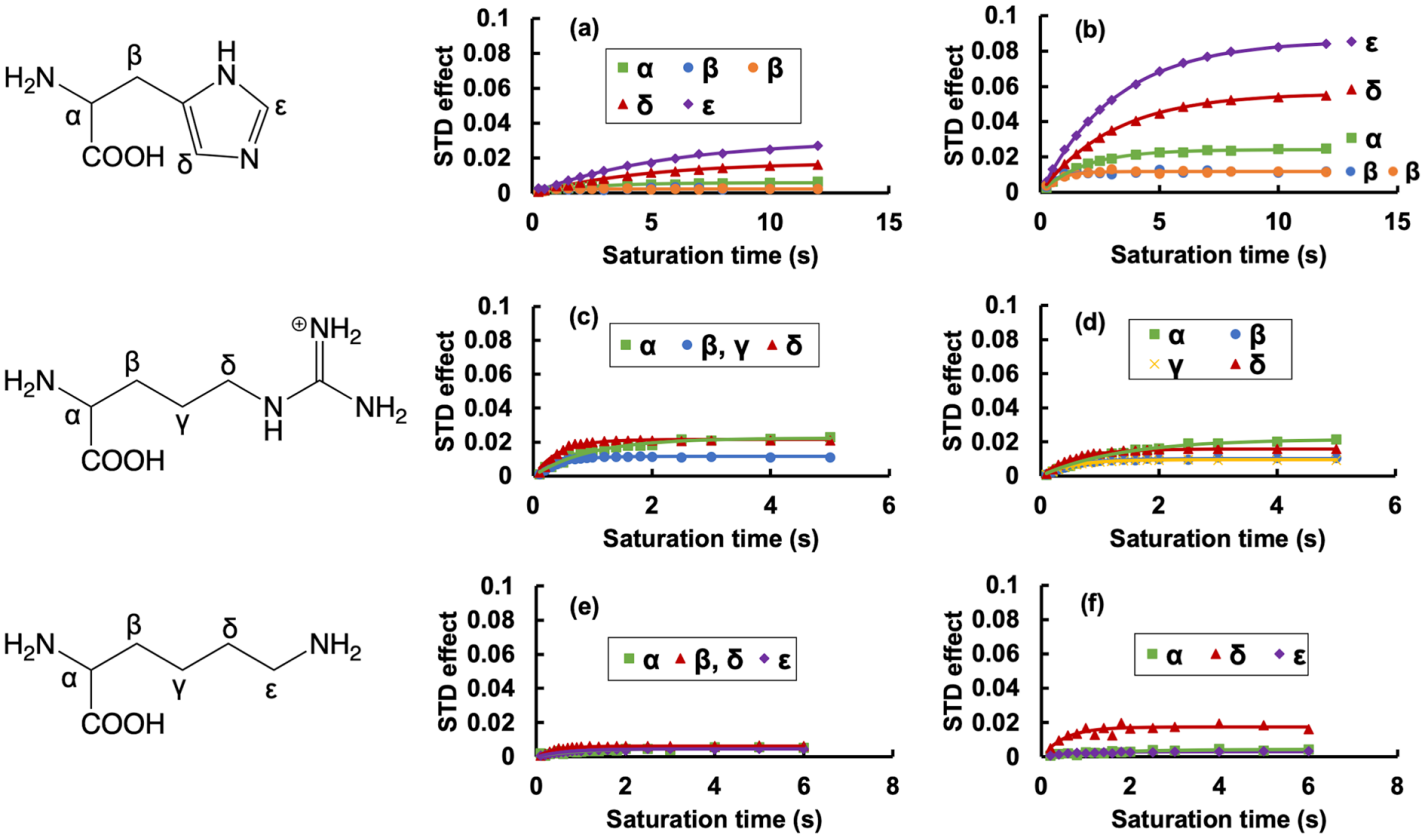
Full STD buildup curves for histidine, arginine, and lysine interacting with aliphatic amine polystyrene beads at high and neutral pH. (**a**,**b**) histidine, (**c**,**d**) arginine, and (**e**,**f**) lysine. The chemical structure of each amino acid with peak assignments is shown to the left of the corresponding plots. (**a**,**c**,**e**) high pH (around pH 9), (**b**,**d**,**f**) neutral pH (around pH 6).

From Fig. 2, we see that the polystyrene beads have a positive charge at low pH, and a negative charge at neutral and high pH. The amino acids histidine, arginine, and lysine are all also positively charged at low pH (with a net ionic charge greater than + 1) and exhibit no binding interaction with the positively charged beads at low pH. At high and neutral pH, the behavior is more complicated, so we display the full STD buildup curves in Fig. 3.

At neutral pH (Fig. 3b,d,f), the polystyrene beads carry a negative charge, while these three amino acids are all positively charged. Although histidine has the lowest net ionic charge at this pH, histidine protons exhibit the largest STD effects at neutral pH. This may be due to the aromatic nature of histidine, and pi–pi interactions between the histidine and styrene molecules of the nanoparticles may also be contributing to binding in addition to electrostatic effects. All three positively-charged amino acids exhibit measurable STD effects when binding to the negatively-charged beads at neutral pH, as expected.

At high pH (Fig. 3a,c,e), the polystyrene beads carry a negative charge, as do histidine and lysine. Since the beads and amino acids have the same charge, electrostatic repulsion decreases the binding strength and the STD effect is reduced. In the case of arginine, at high pH the net ionic charge is only slightly negative (essentially neutral). Therefore, upon going from neutral pH (~ 6.5) to high pH (~ 10) the STD buildup curves of arginine do not significantly decrease, and in fact the initial slope of the buildup curve of the δ proton actually increases. The lack of a substantial difference in the STD buildup curves of arginine between neutral and high pH may be due to hydrophobic effects, which are also present. The difference in attraction between negatively charged beads and positively charged arginine and negatively charged beads and neutral arginine may be offset by the difference in the stability of positively charged versus neutral arginine in water solvent. At neutral pH, positively charged arginine will be more stable in the solvent than neutral arginine at high pH. The destabilizing effect of arginine-water interactions at high pH will drive higher binding of arginine to the polystyrene beads through the hydrophobic effect. This increase in binding at high pH due to the hydrophobic effect may offset the decrease in binding due to reduced electrostatic effects between arginine and the polystyrene beads at neutral pH. This offset leads to approximately equal binding affinity of arginine and aliphatic amine polystyrene beads at neutral and high pH.

**Figure 4:**
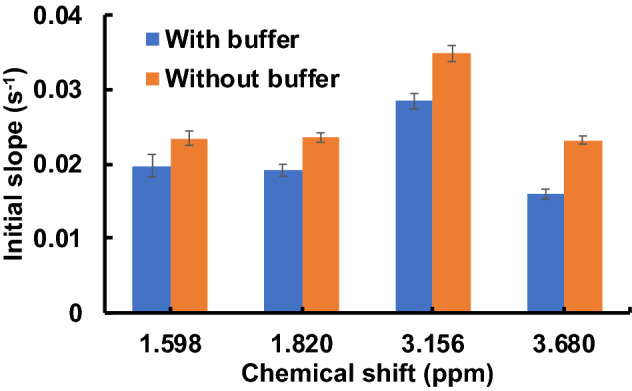
Initial slope of the STD buildup curve of 35 mM arginine interacting with aliphatic amine polystyrene NPs. The blue bars are initial slopes for a sample at pH 6.64 controlled with 200 mM phosphate buffer and the orange bars are initial slopes for a sample at pH 6.31 that was adjusted with 0.1 M HCl.

In order to explore the effects of electrostatic interactions in more detail, in Fig. 4 we compare initial slopes of the STD buildup curve of arginine in samples with similar pH, but different total salt concentrations. Arginine was chosen because at this pH we expect the beads to be negatively charged and the amino acid to be positively charged, and that the interactions between the two are due mainly to electrostatic effects. In order to test this hypothesis, the blue bars in Fig. 4 represent initial slopes for a sample at pH 6.64 that was controlled with 200 mM phosphate buffer, while the orange bars are initial slopes for a sample at pH 6.31 that was adjusted with HCl, and should be expected to have a lower total salt concentration. The high salt concentration of the sample containing buffer will screen the charges on the arginine and polystyrene beads and reduce the electrostatic effects. As expected, this is observed in Fig. 4 as a decrease in the initial slope of the STD buildup curve at high salt concentrations.

**Figure 5:**
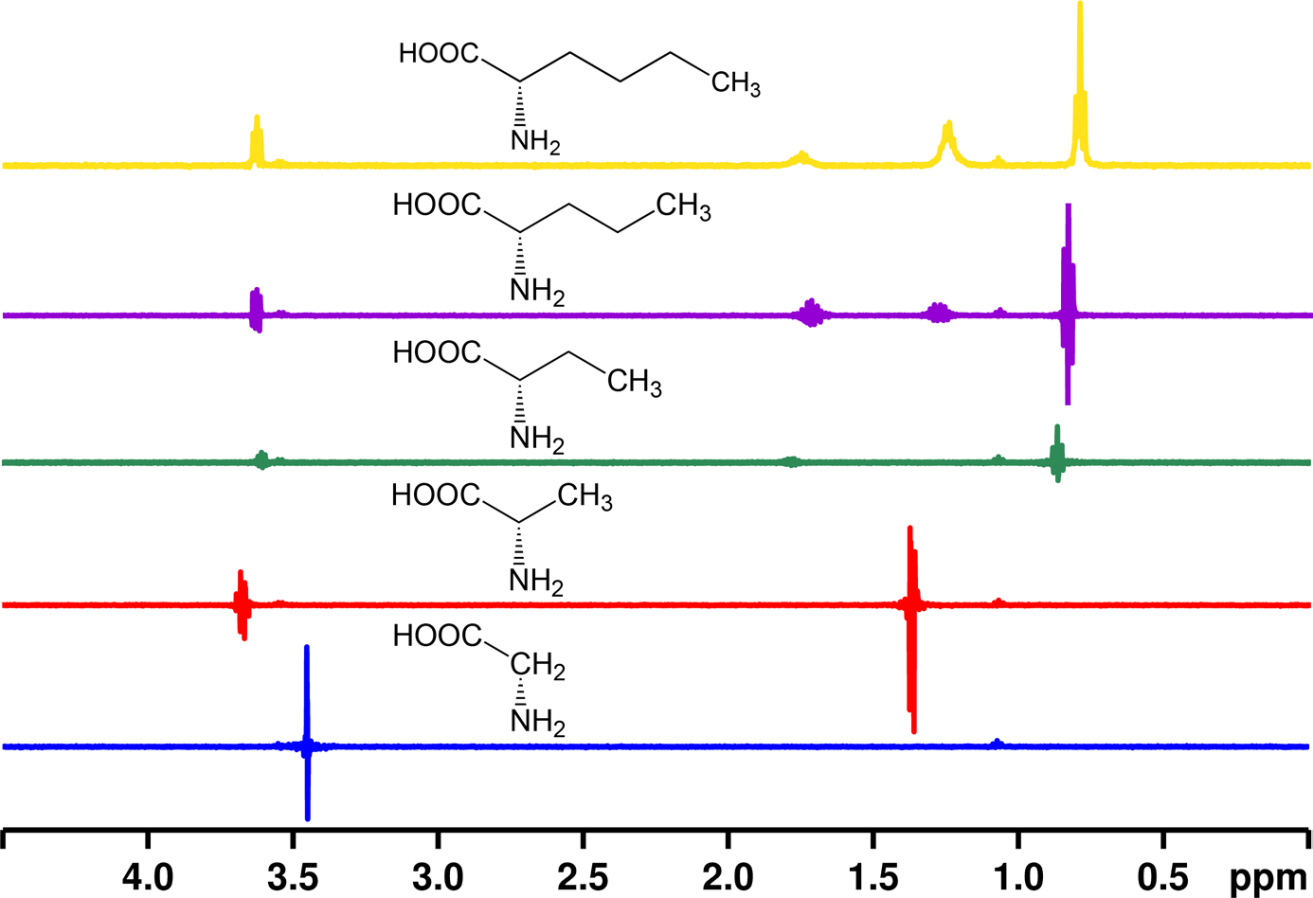
STD difference spectrum of unnatural amino acids with aliphatic amine modified polystyrene nanoparticles at pH 6. From bottom to top, amino acids are glycine, alanine, 2-aminobutyric acid, norvaline and norleucine. The pH was controlled with 200 mM phosphate buffer and specific pH values are listed in supporting information. The concentration of amino acid in each case is 35 mM. The saturation time in each experiment is 10 s.

The last set of amino acids that exhibit observable STD effects when binding to aliphatic amine modified polystyrene nanoparticles as seen in Fig. 1 are long-chain aliphatic amino acids. Other hydrophobic amino acids with shorter side chains, such as alanine and valine, did not exhibit any observable STD effects. In order to explore in more detail the length of the aliphatic side chain that was needed to exhibit measurable binding to polystyrene beads, we performed structure–activity studies on a series of natural and unnatural amino acids with varying lengths of the side chain. The results are shown in Fig. 5. For amino acids with fewer than six carbons, including glycine, alanine, 2-aminobutyric acid, and norvaline, only subtraction errors are observed in the STD difference spectrum (the integral of these signals are close to zero). Of the series of amino acids studied, only norleucine, with a total of six carbons, exhibits an observable STD difference spectrum. The maximum STD effect for norleucine is 0.025. With increasing length of aliphatic side chain, amino acid molecules become more hydrophobic, and thus result in stronger hydrophobic interaction, which acts as the driving force to facilitate the binding to polystyrene nanoparticles.

## Conclusions

In the current work, we obtain more insight into the three modes of binding between amino acids and polystyrene nanoparticles that were proposed in our previous work^31^. By examining binding between charged amino acids and zwitterionic polystyrene beads at different pH values and salt concentrations, we find that electrostatic effects are important to binding. A series of unnatural amino acids with long aliphatic chain substituents were used to establish a structure–activity relationship between the length of the side chain and an observable STD effect.

Based on these studies, we conclude that in order to bind, amino acids must have either a long hydrophobic side chain or aromatic side chain. Once this criterion has been met, electrostatic effects will further contribute to the relative binding intensity of amino acids.

The deeper insight into the relative importance of these three binding interactions will contribute to a better prediction of how other small molecules, peptides, and proteins may interact with the surface of polystyrene nanoparticles.

## Supplementary information

Supplementary Information 1.
