# Supplementary material for: Probing driving forces for binding between nanoparticles and amino acids by saturation-transfer difference NMR
Source: Sci Rep. 2020 Jul 23;10:12351. doi: 10.1038/s41598-020-69185-7 (PMC7378059; doi:10.1038/s41598-020-69185-7)
Supplement: Supplementary file 1 — Supplementary Information 1. [file 41598_2020_69185_MOESM1_ESM.docx]

Supporting Information for:

**Probing Driving Forces for Binding Between Nanoparticles and Amino Acids by Saturation Transfer Difference NMR**

Hui Xu and Leah B. Casabianca

*Department of Chemistry*

*Clemson University, Clemson, SC 29634 USA*

Table S1. pH Values of Each Amino Acid-Polystyrene Bead Sample

Tyrosine was not tested due to the low solubility. Asp and Glu were not tested at pH 6 due to the low solubility. For the Asp and Glu samples at high pH, two drops of 1M NaOH was added to the samples in order to achieve the desired pH, so the concentration of these samples was slightly lower than 35mM.

Table S2. Maximum initial slope values of the STD buildup curve of all amino acids that show significant STD effect at three pH ranges, low pH, neutral pH and high pH. Errors listed are the propagated errors of the fitting error of S_max_ * k.

Table S3. pH values of amino acid in polystyrene nanoparticle sample

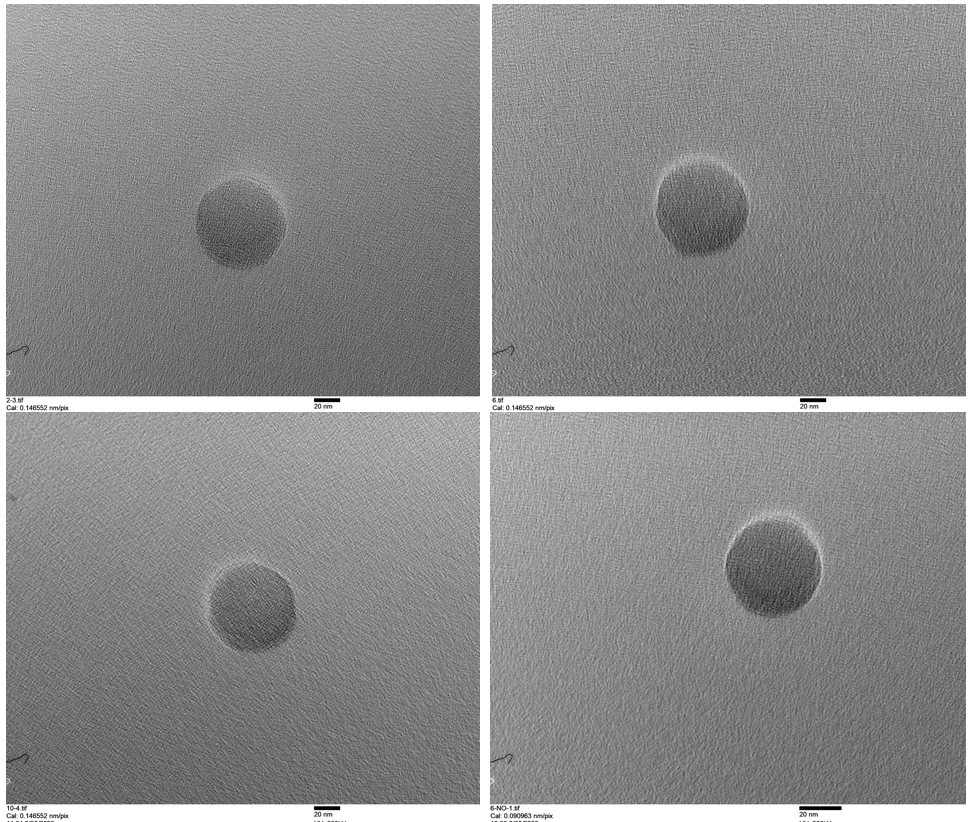


(a)

(b)

(c)

(d)

***Figure S1.*** TEM images of polystyrene nanoparticles at different pH and salt concentrations. 10uL polystyrene nanoparticles are mixed with 0.99mL 200mM phosphate buffer at pH 1.62 (a), 6.09 (b) and 10.48 (c). For sample (d), 10uL nanoparticles are mixed with 0.99 mL de-ionized water, and the pH 6.38 is adjusted with NaOH and HCl. Transmission Electron Microscopy (TEM) experiments were carried out on a Hitachi H-9500 high-resolution transmission electron microscope at 300 kV accelerating voltage. The scale bars are all 20 nm.
